# Moyamoya Disease May Mimic Multiple Sclerosis?

**DOI:** 10.1155/2019/1276950

**Published:** 2019-05-02

**Authors:** Ioanna Spanou, Maria-Eleftheria Evangelopoulos, Georgios Velonakis, Nikolaos Logiotatos, Achilleas Chatziioannou, Constantinos Potagas, Constantinos Kilidireas, Sophia Vassilopoulou

**Affiliations:** ^1^Department of Neurology, Demyelinating Diseases Unit, Eginition Hospital, National and Kapodistrian University of Athens, Greece; ^2^Research Unit of Radiology and Medical Imaging, University of Athens Medical School, Athens, Greece; ^3^Department of Radiology, Areteion Hospital, National and Kapodistrian University of Athens, Greece; ^4^Department of Neurology, Stroke Unit, Eginition Hospital, National and Kapodistrian University of Athens, Greece

## Abstract

**Introduction:**

A wide range of medical conditions may mimic multiple sclerosis. Among them, cerebrovascular diseases, including moyamoya disease, need to be excluded since they share common clinical features and radiographic findings with multiple sclerosis.

**Case Report:**

A 44-year-old woman experienced transient numbness of her right sided face and arm and was referred to our unit due to small brain lesions in magnetic resonance imaging, with a possible diagnosis of multiple sclerosis. Neurological examination was unremarkable except for plantar reflexes and jerky deep tendon reflexes. Brain magnetic resonance angiography revealed findings typically seen in moyamoya disease, confirmed with digital subtraction angiography. Antiplatelet therapy started, but few days later, she developed suddenly global aphasia and right hemiparesis (National Institutes of Health Stroke Scale/NIHSS 6). Brain magnetic resonance imaging revealed acute infarct in the distribution of the left middle cerebral artery. At her discharge, she was significantly improved (NIHSS 3).

**Conclusion:**

Diagnosis of multiple sclerosis is often challenging. In particular, in young patients with transient neurological symptoms and atypical white matter lesions in magnetic resonance imaging, cerebrovascular disorders such as moyamoya disease should be considered in the differential diagnosis. Detailed clinical and neuroimaging evaluation are mandatory for the correct diagnosis.

## 1. Introduction

Multiple sclerosis (MS) is an immune mediated demyelinating disease of the central nervous system. MS has a female preponderance and its diagnosis relies on clinical symptoms and/or the presence of multiple white matter lesions in the magnetic resonance imaging (MRI) demonstrating dissemination in space and time [1, 2]. A wide spectrum of medical conditions can clinically and radiologically mimic MS and should be excluded prior to MS diagnosis and treatment initiation [3]. Considering the high prevalence of MS in females [4] and the atypical lesions at the early stages of the disease, a young female presenting with transient neurological symptoms and small white matter lesions in brain MRI is suspected to be suffering from MS until proved otherwise.

Moyamoya disease (MMD) represents a rare chronic occlusive cerebrovascular disease of unknown etiology characterized by bilateral steno-occlusive changes at the terminal portion of the internal carotid artery (ICA) and an abnormal vascular network at the base of the brain originating either from the basilar artery or extracranial blood vessels [5]. MMD is more common in Asia than in the western countries and only few studies on European populations have been published [6–8]. MMD shares some common clinical and radiological features with MS and needs to be considered in the differential diagnosis, as MMD has a different diagnostic and therapeutic algorithm [9]. This case highlights the necessity of thoroughly examining patient's history and neuroimaging findings in the work-up of suspected MS.

## 2. Case Report

We report a 44-year-old previously healthy woman a month ago, who experienced a transient numbness of her right sided face and arm. She was referred to our unit with a possible diagnosis of MS or of a systemic autoimmune disease due to small brain lesions in MRI. For years, she had been complaining of intermittent weakness of her left arm, but it had been attributed to psychosomatic problems.

Upon presentation, the neurological examination revealed indifferent plantar reflexes and jerky deep tendon reflexes. Her brain MRI revealed various lesions of hyperintensity in T2 and Fluid Attenuated Inversion Recovery (FLAIR) sequence, in the subcortical white matter of the hemispheres and also periventricular, with mild linear cortical, enhancement of the left parietal lobe. Cerebrospinal fluid analysis and laboratory tests for inflammatory-autoimmune diseases were normal.

**Figure 1:**
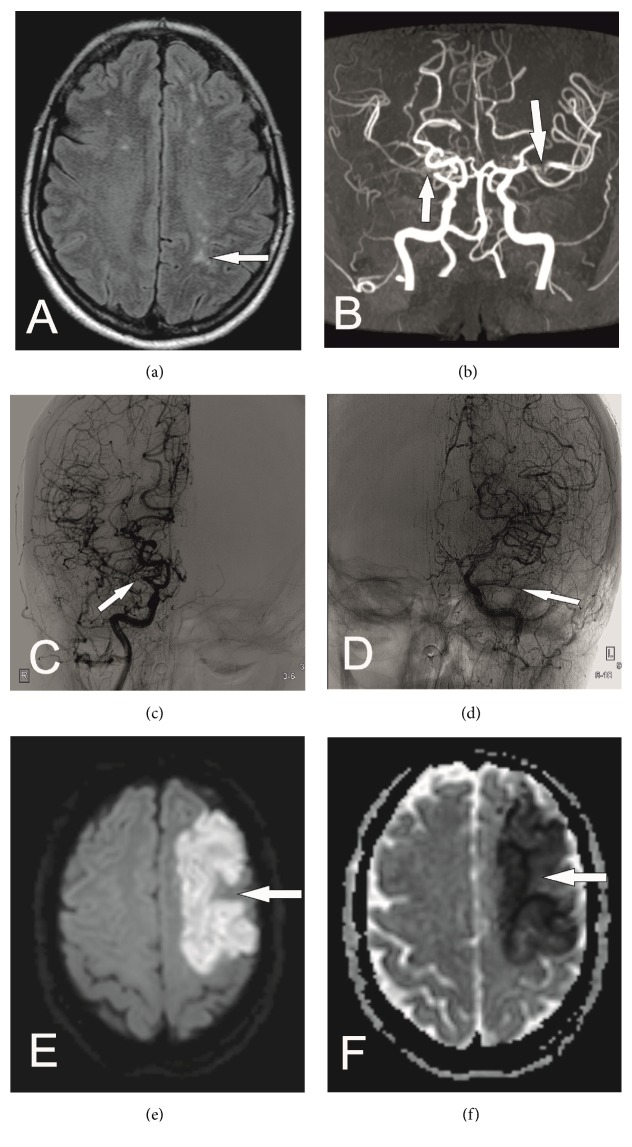
(a) Axial FLAIR image reveals multiple hyperintensities in watershed territories. (b) Time of Flight MRA shows bilateral MCA occlusions (arrows). (c, d) DSA of the right (c) and left (d) internal carotid arteries verifies the MCA occlusions (white arrows). Proliferation of small abnormal net-like vessels is also noticed. Diffusion Weighted Imaging (e) and Apparent Diffusion Coefficient (ADC) map (f) reveal diffusion restriction in the left MCA territory due to an acute infarct (arrows).

However, a more thorough analysis of brain MRI revealed that lesions were mainly in the border zone of anterior cerebral artery (ACA) and middle cerebral artery (MCA) with no evidence of restricted diffusion (Figure 1(a)). Given the watershed distribution of brain lesions, further evaluation with magnetic resonance angiography (MRA) of the brain was recommended to exclude possible cerebrovascular disease. Brain MRA showed a significant stenosis in the proximal segment of MCA and ACA bilaterally and collateralization mainly around M1 and A1 segments, findings typically seen in MMD (Figure 1(b)). Digital subtraction angiography (DSA) of neck and head confirmed diagnosis (Figures 1(c) and 1(d)). Antiplatelet therapy started, but two days later, she suddenly developed global aphasia and right hemiparesis (National Institutes of Health Stroke Scale/ NIHSS 6) and brain MRI revealed acute infarct in the distribution of the left MCA (Figures 1(e) and 1(f)). Thrombolysis was not performed due to increased risk of hemorrhage in MMD and the gradual improvement of the patient. At discharge, she improved with a residual aphasia and right facial paresis (NIHSS 3).

## 3. Discussion

MMD should be considered in the differential diagnosis of MS, as both affect young adults, cause intermittent neurological symptoms, and show multifocal abnormalities on brain imaging [10]. Despite the fact that MMD may have an insidious course, the disease presents with distinct clinical and radiological features that allow the differentiation of these two entities.

The clinical presentations of MMD include transient ischemic attacks (TIAs), ischemic stroke, hemorrhagic stroke, seizures, headache, and cognitive impairment [11]. Few published studies support the theory of a distinct western phenotype in MMD, with female preponderance, lower rates of hemorrhages in adults, and lower evidence of familial occurrence [12]. TIAs in MMD are of paroxysmal onset and typically resolve within 24 hours whereas MS exacerbations tend mainly to evolve subacutely over days and to resolve over days-to-weeks [13]. The anterior circulation is predominantly involved in MMD, and the most frequent ischemic symptom is hemiparesis, followed by speech disturbances and hemisensory abnormalities [14], while in MS, symptoms and signs may depict sensory, motor symptoms as well as brainstem, cerebellum, optic nerve, and spinal cord involvement [13].

Brain MRI can show lesions of T2 hyperintensity in the subcortical cerebral white matter in both disorders. Particularly, in MMD, lesions are especially localized in the frontal and parietal watershed regions and may also affect the deep or superficial grey matter. Additionally, in acute lesions restricted diffusion is particularly common in watershed areas [15]. On the other hand, in MS lesions typically are ovoid, involve the periventricular regions, corpus callosum, brainstem, and cerebellum, may also have a juxtacortical localization, and often demonstrate gadolinium enhancement [16].

The key to diagnosis of MMD is imaging of intracranial vasculature. Definitive diagnosis requires catheter angiography in unilateral cases, while bilateral cases can be promptly diagnosed by either catheter angiography or MRA [17].

## 4. Conclusions

Diagnosis of MS is demanding and several disorders should be considered in the differential diagnosis. In young patients with transient neurological symptoms suggestive of MS and atypical white matter lesions in MRI, vasoocclusive diseases such as MMD should be taken seriously into account in the differential diagnosis. Therefore, increased awareness is required, and detailed clinical and neuroimaging evaluation are mandatory, in order to prevent misdiagnosis and treatment delay.
